# Physiological Stimulation of the Synthesis of Preelastic Fibers in the Dermis of a Patient with Fibrosis

**DOI:** 10.1155/2021/2666867

**Published:** 2021-12-30

**Authors:** Jose Maria Pereira de Godoy, Lívia Maria Pereira de Godoy, Maria de Fatima Guerreiro Godoy, Dalisio de Santi Neto

**Affiliations:** ^1^Adjunct Cardiology and Cardiovascular Surgery Department of the Medicine School, São José Do Rio Preto (FAMERP), CNPq (National Council for Research and Development), São Paulo, Brazil; ^2^Dermatology, Instituto Lauro de Souza Lima-Bauru-Brazil, Member of Researcher Group, Clínica Godoy, São Paulo, Brazil; ^3^Medicine School, São José Do Rio Preto (FAMERP) and Research Group, The Clínica Godoy, São Paulo, Brazil; ^4^Hospital de Base- Medicine School, São José Do Rio Preto (FAMERP), São Paulo, Brazil

## Abstract

**Objective:**

The aim of the present study was to report the physiological stimulation of the synthesis of preelastic fibers in the dermis of a patient with fibrosis.

**Design:**

A clinical study was conducted involving the analysis of histological changes in preelastic fibers following treatment for stage II primary lymphedema for the clinical reversal of lymphedema and fibrosis. *Setting*. University Hospital of the São Jose do Rio Preto of School of Medicine in 2020. Participant was a 67-year-old male patient with late-onset primary lymphedema diagnosed 12 years earlier. Intervention is the lymphatic stimulation using the Godoy method adapted to the treatment of fibrosis. Main outcomes and measures are biopsies before and after treatment. Ten randomly selected histological fields were evaluated using the multipoint morphometric method. The values with this method are relative and expressed as percentages. Statistical analysis was performed with the *t*-test, considering a 95% significance level.

**Results:**

A visible, significant difference in the percentage of preelastic fibers was found between the preintervention and postintervention slides, which were confirmed by the microscopic evaluation and quantification (4.95 ± 0.64% and 14.70 ± 1.06%, respectively).

**Conclusion:**

The physiological stimulation of the lymphatic system using a specific method resulted in the clinical reduction of fibrosis, the return of the elasticity of the skin, and the stimulation of the synthesis of preelastic fibers.

## 1. Introduction

Fibrosis results from conditions that lead to the excessive deposition of extracellular matrix (ECM), compromising tissue function [1]. The main characteristic of fibrosis is the excessive secretion of connective tissue fibers, which is associated with a myriad of diseases that lead to organ failure [2]. The critical event in the development of fibrosis is the aberrant, sustained activation of fibroblasts in myofibroblasts, which produce high levels of ECM, leading to the formation of a dense fibrous tissue in the affected area and increasing local rigidity of the tissue [3, 4].

Diseases with fibrosis place a substantial burden on the healthcare system and economy and are often fatal. Although fibrosis is traditionally considered an irreversible process, there is a growing body of evidence demonstrating that the fibrosis of organs can be reversed under certain circumstances, especially if the underlying cause is removed [5].

Elastic fibers are essential components of all elastic tissues in mammals, such as blood vessels, lungs, and skin, and are extremely important due to the mechanical properties these fibers confer to tissues [6]. The network of dermal fibers is the main effector of elasticity in the skin, enabling it to stretch and return to its original position many times throughout life [7]. Fibrillin-rich microfibrils (FRMs) are integral components of the elastic fiber network, with differential deposition in the papillary dermis in individuals of different skin colors [7].

It is important to note that the resolution of fibrosis requires the degradation of other ECM components, such as proteoglycans [8], elastic fibers, and collagen [9, 10]. Novel concepts in the treatment of lymphedema have been adapted to the treatment of fibrosis. Such concepts propose the normalization or near normalization of lymphedema in all clinical stages, including elephantiasis, with the clinical reversal of fibrosis [11–15]. The aim of the present study was to report the physiological stimulation of the synthesis of preelastic fibers in the dermis of a patient with fibrosis.

## 2. Case Reports

A 67-year-old male patient with late-onset primary lymphedema diagnosed 12 years earlier and one episode of erysipelas in this period was submitted to physical examination, which revealed fibrosis of the dermis characterized by the absence of Godet's sign. The patient was otherwise healthy, not obese, had no comorbidities, and continued working normally. The Godoy method for the treatment of lymphedema [11–15] adapted to fibrosis was proposed to achieve the normalization of the dermis (return of elasticity). The intensive treatment method consists of cervical lymphatic therapy using the Godoy method (approximately 30 gentle movements on the skin in the supraclavicular region 15–20 minutes per day) [12], combined with 8 hours of mechanical lymphatic therapy involving an electromechanical device that performs approximately 25 passive plantar flexion and extension movements per minute [15], 2 hours per day of manual lymphatic therapy [13], and a compression mechanism (hand-crafted stocking made with grosgrain fabric [14] alternated with medium-stretch elastic bandages maintained throughout the entire treatment). These therapies were performed simultaneously totaling about 8 hours/day. The duration of treatment was 2 months, when the clinical reversal of fibrosis was achieved, and the elasticity of the skin had improved. The clinical evaluation of the skin was constant throughout the treatment, and at the time, it had its elasticity clinically evaluated by manual skin pinching. At that time, there was no difference in this elasticity in relation to the normal contralateral limb. At this point, the postintervention biopsy was performed. Biopsies were performed before and after the intervention in the same region at a distance of 1 cm of other in parallel. For such, basic surgical care was taken, such as asepsis and antisepsis, local anesthesia using 2 ml de xylocaine 2%, followed by a longitudinal wedge-shaped incision approximately 1 cm in length and 0.5 cm in width. The biopsied material was maintained in 10% formol and embedded in paraffin. The slides were stained with orcein and resorcin-fuchsin evaluated under an optical microscope. The magnifications were 10X and 40X. Ten randomly selected histological fields were evaluated using the multipoint morphometric method proposed by Weibel [16]. The values with this method are relative and expressed as percentages. The data were submitted to a normality test and expressed as mean and standard deviation values. Comparisons were performed using the *t*-test with the level of significance set at 95%, using Stats direct 3.

**Figure 1:**
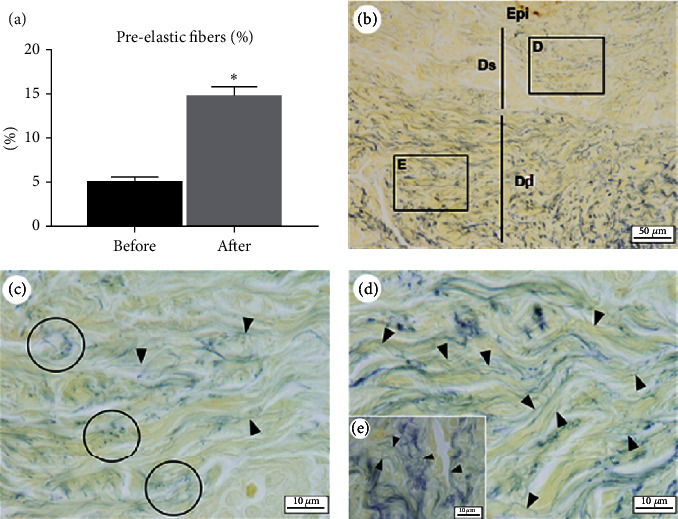
Preelastic fibers. (A) Graph showing a significant difference in percentage of preelastic fibers after treatment for lymphedema. (B, D, E) Dermis after treatment—note increase in preelastic fibers in superficial dermis (shown in (D)) and increase in number and thickness of fibers in deep dermis (shown in (E)). (C) Dermis before treatment—note rare preelastic fibers and regions of breakage of elastic fibers (circles). Epi, epidermis; Ds, superficial dermis; Dd, deep dermis. Arrowhead, preelastic fibers; stain, orcein and resorcin-fuchsin.

A visible, significant difference in the percentage of preelastic fibers was found between the preintervention and postintervention slides, which was confirmed by the microscopic evaluation and quantification (4.95 ± 0.64% and 14.70 ± 1.06%, *p* value < 0.05, respectively) (Table 1 and Figure 1).

Lymphedema has no cure; however, it is possible to normalize the edema and maintain the results by performing maintenance treatment in a partial or total way. In this case, use the grosgrain stocking.

This study received approval from the Institutional Review Board of the São Jose do Rio Preto School of Medicine (certificate number: 4.398.518). The patient signed a statement of informed consent.

## 3. Discussion

This is the first study in the international literature to show the possibility of the physiological stimulation of the synthesis of preelastic fibers and remodeling of these fibers during the clinical treatment of primary lymphedema with fibrosis. In a previous evaluation (in publication phase), an important rearrangement of elastin was found, with an approximately 250% increase in the number of fibers. In the present investigation, a more specific evaluation was performed of preelastic fibers in the dermis, in which a significant increase of 336% was found after treatment, with an increase in the number of fibers in the superficial dermis as well as an increase in the number and thickness of fibers in the deep dermis.

The treatment method influenced the physiological remodeling of this protein, which could be attributed to the synthesis of new elastic fibers. These fibers account for only 2–4% of the ECM but play an important role in the elasticity of the skin [14]. The present study paves the way for a new line of research involving the physiological stimulation of the production and remodeling of elastin.

The treatment method employed is a novel concept for the treatment of lymphedema adapted to fibrosis, which has specific characteristics. The method is proposed to achieve the clinical reversal of lymphedema, including elephantiasis, to standards of normality or near normality [11].

The main hypothesis for the development of fibrosis in lymphedema is the lack of the mobilization of macromolecules by the lymphatic system combined with other mechanisms, such as inflammation, interference in the production of the elements of the ECM, and the failure of fibrinolysis. All these mechanisms are currently being evaluated specifically to gain a better understanding of these cellular and ECM dynamics.

Elastin and collagen are the main constituents of the fibers of the ECM and are therefore of fundamental importance in the development of fibrosis. Further studies are needed to identify each of the structures and mechanisms that may be involved in the dynamics of the formation of fibrosis, which has different stages and each stage may have distinct characteristics. This study was presented at the World Congress of Lymphology in Greece in 2021 [17, 18].

## 4. Conclusion

The physiological stimulation of the lymphatic system using a specific method resulted in the clinical reduction of fibrosis, the return of the elasticity of the skin, and the stimulation of the synthesis of preelastic fibers.

## Figures and Tables

**Table 1:** Morphometry of epidermis and dermal papillae and area of connective fibers before and after treatment.

	Before	After
Preelastic fibers (%)	4.95 ± 0.64^b^	14.70 ± 1.06^a^

^a,b^Different superscript letters denote significant difference between evaluation times (*p* < 0.05).

## Data Availability

The data (statistic analysis) used to support the findings of this study are included within the article.
